# Tunisian general practitioner’s perception of benzodiazepine prescription:

**DOI:** 10.1192/j.eurpsy.2023.714

**Published:** 2023-07-19

**Authors:** S. Boudriga, A. Aissa, A. Benzarti, Y. Zgueb, U. Ouali, R. Jomli

**Affiliations:** Departement psychiatry A, Razi Hospital, Tunis, Tunisia

## Abstract

**Introduction:**

Despite the scientific requirements and restrictive recommendations, there is a significant disparity between theory and practice in the prescription of benzodiazepine(BZD). Long-term prescribing, defined by a duration exceeding six months has been commonly reported worldwide, some authors explained this by physician’s perceptions.

**Objectives:**

This study aimed to evaluate the perception of general practitioners practicing in Tunis, in the private or public sector, concerning the prescription of BZDs.

**Methods:**

A cross-sectional study was conducted among general practitioners in the private and public sectors practicing in Tunis during the study period (September and October 2021). It is based on the response to a questionnaire, which focused on the perception of prescribing BZD, via google forms distributed to members of the regional committee of the order of physicians.

**Results:**

A total of 75 physicians participated in the study. The mean age was 47.75± 12,2 years, with 17,28 ± 11,8 years of clinical experience. Among the 75 participating physicians, 83% considered that patients on BZD patients had a better quality of sleep and 58% assert that patients on BZDs had restful sleep. 83% of participants agreed that BZDs were associated with fewer nocturnal awakenings 83% and 85% with a decrease in the feeling of irritability. 18% of the doctors think that the easiest way to manage a patient’s anxiety is to prescribe a BZD. 24 doctors believe that chronic use of BZDs is essential to control anxiety. patients’ anxiety. The number of years of practice is inversely correlated with the perception that the patient wakes up less at night (p= 0.059). Male gender correlates with the perception that it is acceptable to continue prescribing beyond the recommended duration as long as they are well tolerated (p=0.035).

**Conclusions:**

BZD prescription decision in general medicine is complex. This study participates in increasing our level of understanding of the reasons behind the long-term prescription of this molecule.

**Disclosure of Interest:**

None Declared

